# Antimicrobial Properties of Polyaniline and Polypyrrole Decorated with Zinc-Doped Copper Oxide Microparticles

**DOI:** 10.3390/polym12061286

**Published:** 2020-06-04

**Authors:** Moorthy Maruthapandi, Arumugam Saravanan, John H. T. Luong, Aharon Gedanken

**Affiliations:** 1 Bar-Ilan Institute for Nanotechnology and Advanced Materials, Department of Chemistry, Bar-Ilan University, Ramat-Gan 52900, Israel; lewismartin.jesus@gmail.com (M.M.); saran.bc94@gmail.com (A.S.); 2 School of Chemistry, University College Cork, Cork T12 YN60, Ireland; luongprof@gmail.com

**Keywords:** Polypyrrole, zinc-doped copper oxide, polymer composite, antimicrobial, *Escherichia coli*, *Staphylococcus aureus*

## Abstract

Polyaniline (PANI) and polypyrrole (PPY) were synthesized by carbon dots (CDs) under UV irradiation and then sonicated together with zinc acetate and copper acetate to form the PANI-Zn@CuO and PPY-Zn@Cu composites. The former consisted of agglomerated spherical particles with diameters of 1–5 µm, whereas the latter displayed irregular stick shapes with similar diameters. The bacterial potency of the composites against *Escherichia coli* and *Staphylococcus aureus* was enhanced remarkably with Zn doping in the CuO matrix, designated as Zn_0.11_Cu_0.89_O, at 0.144 mg/mL. The cell death was mainly attributed to the release of reactive oxygen species (ROS) that would severely damage DNA, proteins, and lipids. Bacteria could adhere to neutral surfaces of the composites by van der Waals attractive forces. The binding event disrupted the native surface charge of bacterial cells to induce cell lysis and result in eventual cell death.

## 1. Introduction

Metal oxide nanoparticles (NPs), particularly ZnO and CuO, have emerged as potential antimicrobial agents to overcome the rising global issue of antimicrobial resistance against conventional antibiotics. Their appealing features include durability and high stability with low mammalian cell toxicity, compared with organic counterparts. ZnO reduces oxygen to water, and this reaction involves the release of three intermediate reactive oxygen species (ROS): superoxide, hydrogen peroxide, and hydroxyl radical [1,2]. Such species play an essential role in eradicating both Gram-negative and Gram-positive pathogens. Copper is a well-known antimicrobial agent, and its action against bacteria is related to Fenton type reactions [3,4,5]. Cupric oxide (Cu=O) is the most widely studied because Cu_2_O and Cu_2_O_3_ are not stable materials. Copper NPs kill bacteria most likely via the release of Cu^2+^ ions [6]. Albeit, the mechanism of copper in cell death is not fully understood, and bacterial resistance to copper particles has been noted [7]. The combined antimicrobial properties of ZnO and CuO can be realized by doping copper in a ZnO matrix [8] or doping zinc in a CuO matrix [9]. It becomes a formidable task for bacteria to develop strong resistance pathways against such hybridized metal particles [10]. However, NPs suffer from particle instability and degradation, resulting in reduced NP efficacy or longevity. In aqueous media, the use of NPs is complicated by their propensity toward agglomeration to reduce the active surface area, the key parameter of their interaction with bacteria. Thus, polymer composites prepared from conducting polymers and metal NPs might be a solution to circumvent these shortcomings.

Among several conducting polymers, polyaniline (PANI) and polypyrrole (PPY), have received considerable interest because of their unique properties and ease of preparation by chemical or electrochemical methods. PANI and PPY can also be easily prepared using carbon dots (CDs) or nitrogen-doped CDs (N@CDs) as an effective initiator [11,12]. Of importance is the pioneering synthesis and applications of Cu_0.89_Zn_0.11_O (marked here as Zn@CuO, MW = 79.75) as an antibacterial agent [13,14]. Hybridized metal oxide nanomaterials, e.g., Zn-doped CuO (Zn@CuO), are of specific interest, owing to their size-dependent biological, optical, and electrical properties. To date, PPY, PANI, and other polymers are synthesized and decorated with metals or metal oxide nanoparticles and used as antibacterial agents (Table 1). However, these polymer composites have several drawbacks such as complex synthesis, toxicity, and the high cost of noble metals (Au, Pt, Ag, and Pd).

This study aims to synthesize and evaluate the antibacterial activities of two polymers decorated with Zn@CuO microparticles. PANI and PPY are polymerized from their corresponding monomers using CDs as an initiator. The prepared Zn-doped CuO is decorated on such polymers via a one-step sonochemical method. The antibacterial effects of the polymer composites are evaluated against *Escherichia coli* (Gram-negative) and *Staphylococcus aureus* (Gram-positive). A mechanism of bacterial death is postulated in corroboration with the experimental data.

## 2. Experimental Section

### 2.1. Preparation of CDs

Polyethylene glycol (40 mL, PEG-400) was transferred into a 50 mL beaker and placed in a water bath at 70 °C. The tip of an ultrasonic transducer was dipped in the PEG-400 and sonicated for 3 h with a 40% amplitude [11,12,25,26].

### 2.2. Preparation of PANI

The reaction mixture consisting of 1.0 g aniline (1.1 mmol), 25 mL of HNO_3_ (2 M), and 3 mL of the CD solution (5 mg CDs) was illuminated with UV light for 48 h. The resulting blackish-brown slurry was filtered, washed with deionized water, and dried at room temperature [11].

### 2.3. Preparation of PPY

Pyrrole (11 mmol, 0.74 g) was mixed with 25 mL of 0.3 M nitric acid and 2.5 mL (8 mg) of CDs. After activation under UV light for two days, the blackish-brown solid obtained by filtration was washed with deionized water and ethanol to remove unreacted CDs and dried at room temperature [11,25,26].

### 2.4. Synthesis of PANI-Zn@CuO and PPY-Zn@CuO

An amount of 0.1 g of PANI (0.11 mmol) or PPY (0.16 mmol) was dissolved in 90 mL of ethanol. The copper acetate and zinc acetate (3:1 molar ratio) salt solution (10 mL) was then added, resulting in a final concentration (0.01 M of 3:1 molar ratio). The reaction mixture was sonicated until the temperature reached 70 °C, and the solution was maintained at pH 8–9 by adding an ammonia solution. The blue-tinted solution turned blackish-brown and was placed in an ice bath and sonicated for another 30 min [26].

### 2.5. Antibacterial Tests

*E. coli* or *S. aureus* were incubated overnight under aerobic conditions at 37 °C in Luria–Bertani (LB) broth. This broth contains 10 mg/mL tryptone (a mixture of peptides prepared from casein), 5 mg/mL yeast extract, 5–10 or 10 mg/mL NaCl, and 1 mg/mL glucose. The bacterial concentrations were related to the absorbance at 595 nm (OD_595_) until the final concentration of 10^7^ cells was attained. For the antibacterial tests, 500 µL of the polymer composite was added to the 500 µL of the bacterial suspension. The mixture was incubated at 37 °C with shaking at 200 rpm. A 100 µL aliquot was taken after 0, 3, and 12 h, and diluted to 10-fold in the 20% LB medium and plated on the nutrient gar plates. After the plates were incubated for 16 h at 37 °C, bacterial colonies at appropriate dilutions were counted to estimate the number of viable bacteria by the colony-forming unit (CFU) method. A colony-forming unit is a unit used in microbiology to estimate the number of viable bacteria in a sample. The visible bacteria were counted by visualization.

### 2.6. Analytical Techniques

FTIR spectra of the polymer composites were measured using a Transon 27 spectrometer (Bruker, Karlsruhe, Germany), whereas their crystalline nature was analyzed using X-ray diffraction (Bruker, AXS D8 Advance diffractometer, Germany). SEM (FEI Magellan 400 L microscope, Hillsboro, OR, USA) was performed to probe the size and morphology of the composites. For SEM analysis, the samples were prepared by placing a small amount of the dried powder on a carbon tape attached to a copper strip, and the material was coated with gold to improve conductivity. ^13^C solid-state nuclear magnetic resonance (NMR) spectra of the composites were acquired using a Bruker 5000 Ultra Shield spectrometer (Bruker, Billerica, MA, USA). The zeta potential was measured using a Malvern Zetasizer Nano-ZS (Malvern, UK).

## 3. Result and Discussion

### 3.1. FTIR Spectra and X-ray Diffraction Pattern

The doping of Zn in the CuO matrix deserves a brief comment, albeit this nanocomposite has been well reported elsewhere [13,14]. Among all transition metals, Zn causes more effective doping, as it has a comparable ionic radius with Cu^2+^ (0.074 vs. 0.072 nm) and possesses the same oxidation states [27,28,29]. Thus, the CuO host lattice might not undergo any significant local structural changes. However, the presence of the Zn^2+^ dopant can effectively produce defects in CuO nanostructures, an appealing feature for biological applications [30]. During the composite formation, Zn@CuO particles interact with the polymers through the chemical bond or polymer chains. Thus, FTIR, XRD, and solid-state NMR were conducted to characterize these two composites [31].

**Figure 1:**
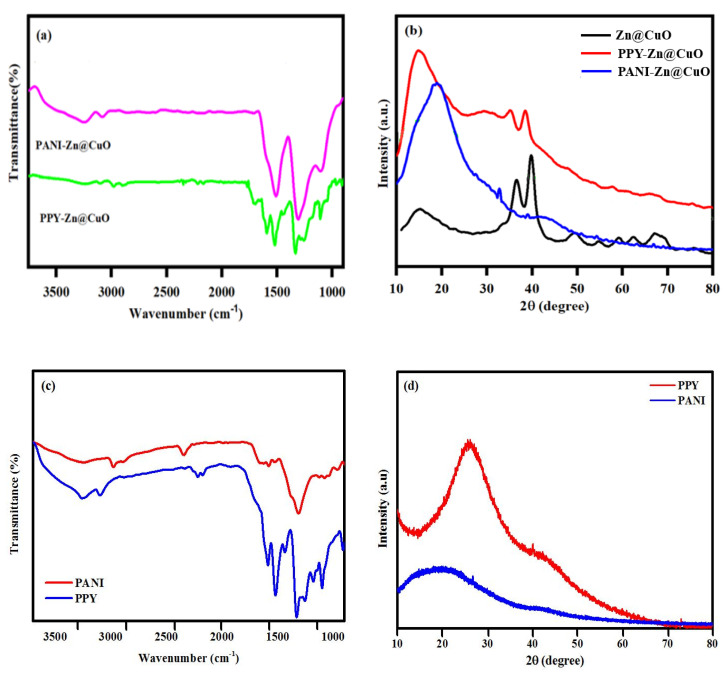
(**a**,**c**) FTIR spectrum and (**b**,**d**) XRD diffraction patterns of polymers and polymers’ macro-nanocomposites. In Figure 1b, the two highest peaks of Zn@CuO are known as (−1, 1, 1) and (1, 1, 1), followed by (2, 0, 2). The identities of other peaks can be found in Reference [28]. A very small peak (1, 1, 0) before the (−1, 1, 1) peak has been reported in the literature, e.g., Reference [27] but only appears as a broad peak in our experimental conditions.

The FTIR spectra of PPY and PANI (Figure 1a–c) exhibit some typical broad bands at 3100–3390 cm^−1^ (N–H stretching) and ~1585–1520 cm^−1^ (C=C stretching). The peaks at 3095 and 2981 cm^−1^ are attributed to the C–H aromatic stretching, whereas the peak at 1330 cm^−1^ is assigned to the C–N stretching. The presence of =C–H in-plane vibrations is confirmed by two peaks at 1120 and 1260 cm^−1^. These similar peaks also appear in the polymer composites as shown in Figure 1a. However, the stretching vibrations of N–H, C=C, C–N, and =C–H of the composites display noticeable peak shifting and reduced peak intensities and form as broader bands. The XRD patterns of the polymers and their composites are shown in Figure 1b,d. The two polymers are amorphous as reflected by a broad peak around 2θ = 10°–40°, a common feature of amorphous compounds. The polymer composites exhibited a broad XRD peak around 2*θ* = 12°–36.6°, due to periodicity parallel to the polymer chain (Figure 1b). A shift of 14.3° confirmed the incorporation of Zn@CuO particles in the polymer chains. The composites also had minor sharp peaks around 2*θ* = 32.1°, 35.1°, and 41.6°, as a typical signature of the Zn@CuO powder. Other small and broad peaks were also noted, in agreement with the literature [13,14]. These peaks were slightly shifted for the PPY composite but disappeared in the PANI composite. These results illustrated the presence of Zn@CuO in the composites and that at least the PPY composites with Zn@CuO are partially crystalline.

### 3.2. Morphology and Elemental Analysis

**Figure 2:**
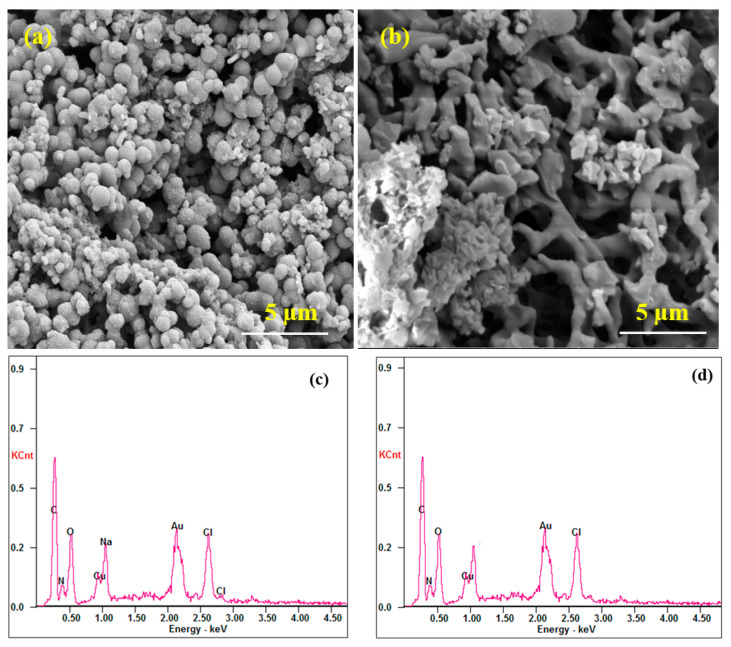
SEM and EDX images of the polymer composites. Notes: (**a**,**b**) SEM of polypyrrole (PPY)-Zn@CuO, polyaniline (PANI)-Zn@CuO; (**c**,**d**) EDX of PPY-Zn@CuO, PANI-Zn@CuO.

SEM micrographs of PANI-Zn@CuO and PPY-Zn@CuO macro-nanocomposites are shown in Figure 2. The PPY-Zn@CuO composites exhibit agglomerated particles with spherical shapes and diameters ranging from 1 to 5 µm. The PANI-Zn@CuO composite displays irregular stick shapes with a broad range diameter from 1 to 5 µm, compared with 20–200 nm of the Zn@CuO NPs’ diameter [13,14]. The EDX spectra and SEM-EDX elemental mapping spectra of the polymer composites show the elementary components (C, N, O, and Au) with uniform distribution. Figure 2d,e also confirmed the formation of the metal oxide of CuO. The SEM-EDX elemental mapping spectra of PPY-Zn@CuO and PANI-Zn@CuO further confirmed the presence of CuO. Zn was not detected by the SEM-EDX, possibly due to its very low percentage in the composites.

### 3.3. Solid-State ^13^C NMR Analysis

**Figure 3:**
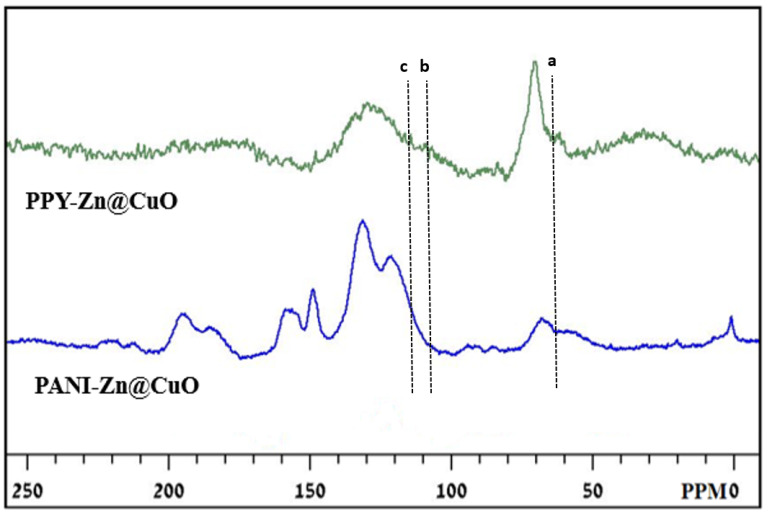
Solid-state ^13^C NMR spectra of PPY-Zn@CuO and PANI-Zn@CuO macro-nanocomposites.

The solid-state NMR spectra of PPY-Zn@CuO and PANI-Zn@CuO exhibit a broad and a sharp peak (Figure 3). The peak at 125 ppm is attributed to the protonated carbon in the polymer chain. The shoulder peaks at 110 and 114 ppm corresponds to the protonated carbons in the aromatic rings and a peak at 124 ppm is related to the non-protonated carbons. The sharp peaks at 61 and 77 ppm are originated from the protonated C-9 and non-protonated C-16 carbon of the quinoid part of the polymer structure. The solid-state 13C-NMR spectra of PPY-Zn@CuO and PANI-Zn@CuO are almost similar compared with those of PANI and PPY. The spectra of composites are slightly shifted towards the upfield with lower shoulder peak intensities (a, b, and c), at 63, 108, and 115 ppm, lower than those of the bare polymers. These variations attest to the formation of a metal oxide composite with the polymer. The ^13^C-NMR spectra of PANI and PPY are available from the literature [11,25,26].

### 3.4. Zeta Potential Measurements

**Figure 4:**
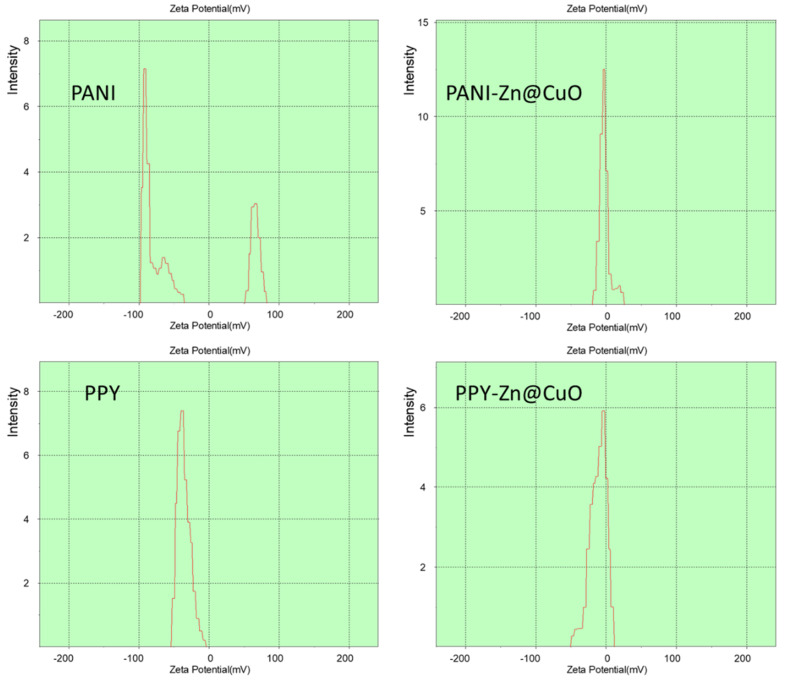
Zeta potentials of PANI, PANI-Zn@Cu-O, PPY, and PPY-Zn@CuO.

The zeta potential of PANI has been known to be pH-dependent and very close to 0 mV but still positive at pH 7 [32], as its isoelectric point (IEP) is reached at pH 8.2 (Figure 4, upper, left). In the cell culture medium, PANI exhibited a negative peak of around −85 mV, compared with ~0 mV for PANI-Zn@CuO (Figure 4, upper). Of notice was the presence of an additional positive peak (Figure 4, upper-left) for the zeta potential of PANI. The nonspecific adsorption of ions or polyelectrolytes onto the PANI or PANI-Zn@CuO surface, however, altered the value and polarity of the measured zeta potential significantly. However, such zeta potentials were still useful to compare the surface charges of PANI and PANI-Zn@CuO. A small positive peak ~ 70 mV could be attributed to the free ions or polyelectrolytes of the LB medium. Similarly, PPY-Zn@CuO displayed an asymmetrical broad peak around 0 mV, compared with −30 mV for PPY (Figure 4, lower). Polypyrrole undergoes protonation or deprotonation reactions in an alkaline or acid solution, resulting in the change of its surface charges (the surface changed between a charged and a neutral state) [33]. Again, a negative charge of PPY could be due to the adsorption of excess negatively charged molecules in the LB cell culture broth.

### 3.5. Antibacterial Activity

**Figure 5:**
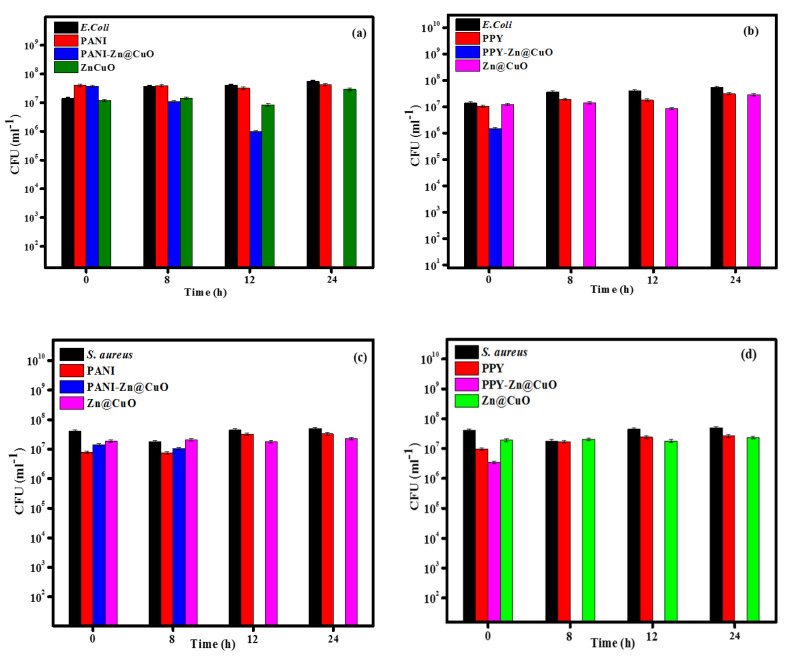
(**a**,**b**) Antimicrobial effects of PANI-Zn@CuO and PPY-Zn@CuO on *E. coli* and (**c**,**d**) antimicrobial effects of PANI-Zn@CuO and PPY-Zn@CuO on *S. aureus*. The concentration of pure PANI or PPY is 1 mg/mL, the concentration of Zn@CuO is 0.144 mg/mL, and the polymer composite consists of 0.8 mg/mL of PANI or PPY together with 0.144 mg/mL of Zn@CuO.

*E. coli* and *S. aureus* were eradicated after 8 h of their exposure to the PANI-Zn@CuO and PPY-Zn@CuO polymer composites at different concentrations to establish the minimum inhibition concentration for each composite. The results revealed a synergetic effect perceived for these composites compared with PANI and PPY and Zn@CuO (Figure 5). The Zn@CuO level in the composites was determined by ICP. The bacterial activity was performed at the minimum composite concentration of 1 mg/mL, comprising 0.144 mg/mL of Zn@CuO. This MIC was a significantly lower amount of Zn@CuO as compared with the literature value of 1 mg/mL of Zn@CuO after 24 h of exposure [9].

The minimal amount of Zn@CuO was of importance considering the plausible cytotoxic effect of metal oxide particles for their applications as antibacterial agents. *E. coli* was eradicated by PANI-Zn@CuO after 24 h of exposure (Figure 5a,b), whereas S. *aureus* was eradicated by the PANI-Zn@CuO and PPY-Zn@CuO composites after 12 and 8 h of exposure, respectively (Figure 5c,d). A noticeably shorter time of 8 h was observed when *E. coli* or *S. aureus* were exposed to the PPY-Zn@CuO composite (Figure 5e,f).

The cell surface of most bacteria has a moderately negative net charge at neutral pH [33]. Gram-positive bacteria possess a negative charge due to the presence of teichoic acids linked to the peptidoglycan or the underlying plasma membrane. The lipopolysaccharides impart a strong negative charge to the surface of Gram-negative bacterial cells. The zeta potentials of 22 *E. coli* strains were negatively charged in the 150 mM PBS (pH 7.4), ranging from −5 to −34 mV [34]. Both PANI and PPY with negative charges exhibited no antibacterial effect against *E. coli* and *S. aureus*, plausibly due to the much stronger net negative charge of PANI and PPY. A pronounced repulsive force between the bacteria and the polymers circumvented any adherence of the bacteria to the polymers.

### 3.6. A Postulated Mechanism of Cell Death

**Scheme 1:**
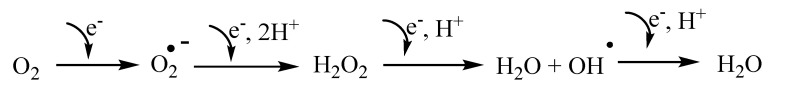
The reduction of oxygen to form water by Zn@CuO, which is involved in the release of superoxide anions, hydrogen peroxide, and hydroxyl radicals.

**Figure 6:**
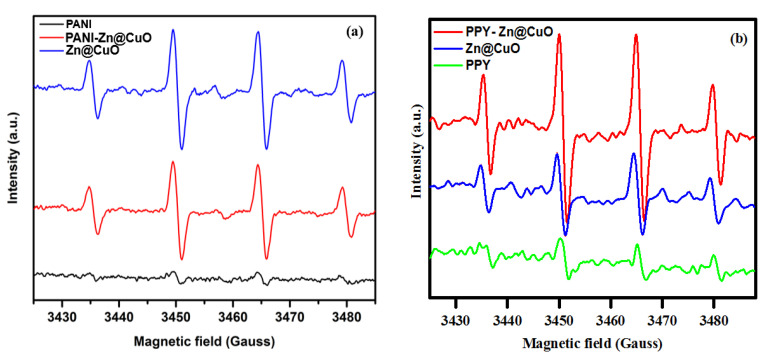
Electron paramagnetic resonance (EPR) measurement for (**a**) PANI-Zn@CuO and (**b**) PPY-Zn@CuO.

CuO [35] and ZnO [36] are known to generate reactive oxygen species (ROS) to eradicate both Gram-negative and Gram-positive bacteria. The release of reactive oxygen species (ROS) is expected to impair bacterial membranes, DNA, and proteins. Zn@CuO can generate ROS on its surface and this phenomenon is enhanced by excitation with UV light [37,38]. CuO NPs are also known to generate ROS to modify bacterial molecules and block the antioxidant defense system [39]. More defective sites are anticipated on the Zn@CuO surface compared with that of CuO due to the doping of Zn [28]. Thus, more oxygen is adsorbed on such defective sites of Zn@CuO, leading to more ROS on its surface as this hybridized metal oxide is capable of reducing oxygen to form water as follows (Scheme 1):

The effect of oxygen stress was confirmed by using the electron paramagnetic resonance (EPR) technique to measure the ROS generation with DMPO (5,5-dimethyl-1-pyrroline-*N*-oxide) as a spin trap [6]. DMPO captures hydroxyl (OH**·**) radicals and superoxide anions (O^2−^) to create DMPO–OH as a final adduct with a distinct quartet signal and a typical 1:3:3:1 signal intensity ratio. The PPY polymer produced more ROS compared with its counterpart, PANI (Figure 6), but was lower than the ROS level of Zn@CuO. Consequently, the ROS level of PPY-Zn@CuO was higher than that of PANI-Zn@CuO. The synergistic effect between PANI and PPY with Zn@CuO was attributed to the free electrons in the polymers, which could be combined with the O_2_ in the suspension to enhance the production of ROS [7]. The ROS include oxygen radicals such as peroxides, superoxide, hydroxyl radical, singlet oxygen, and α-oxygen. They are unstable molecules that readily react with cellular molecules, causing damage to DNA, RNA, and proteins that support cell growth and proliferation. Oxidative DNA damage is known as the primary cause of cell death [40,41]. Notice that the superoxide radical with a negative charge cannot penetrate the cell membrane, i.e., it only acts on the outer surface of bacteria. In contrast, hydrogen peroxide should be able to penetrate the bacterial cell wall to trigger cell plasmolysis. Of note is the presence of bacterial superoxide dismutase and catalases that degrade superoxide and hydrogen peroxide, respectively. However, no cellular detoxification mechanism is known for hydroxyl radicals. With such combined attacks and the release of hydroxyl radicals, the microbes cannot develop new resistant strains. Lipid peroxidation is a signature of ROS damage, as polyunsaturated fatty acids of lipids are susceptible to free radical-initiated oxidation and participate in chain reactions to damage biomolecules required to support cell growth and replication [42]. It was reasoned that Zn doping into the CuO matrix resulted in the formation of structural defects such as oxygen vacancies and Cu interstitial defects, serving as trapping centers for electrons [13,27].

Another plausible mechanism was attributed to the adhesion of bacteria to the composites. The direct interaction of bacteria and surfaces is governed by van der Waals attractive forces, whereas interionic forces are attractive or repulsive [43], even if bacteria and surfaces are charged alike, i.e., negative van der Waals forces can overcome repulsion and lead to adhesion [44]. Therefore, bacteria can adhere to hydrophobic or slightly negatively charged surfaces, e.g., glass and polystyrene. The zeta potentials of the two composites were close to 0 mV, i.e., the ionic interaction is negligible compared with van der Waals attractive forces. The two composites acted to disrupt the native surface charge of the bacterial cells, which induced cell lysis, resulting in cell death.

The cytotoxicity of Zn@CuO deserves a brief mention here because it induces only mild acute toxicity in *Xenopus laevis* embryos, African clawed frog, compared with ZnO [45]. Zn-CuO NPs exert noticeable anti-proliferation effects on cancer cells but these effects are relatively weak on normal cells [46]. The low cytotoxicity of Zn-CuO opens a new route for the development of effective antimicrobial composites with various biocompatible polymers. Of importance is the study of the shape-dependent cytotoxicity of polyaniline nanomaterials in human fibroblast cells [47]. The toxicity increases with the decreasing aspect ratio of the PANI nanomaterials (length/diameter ratio: 2.09, 2.94, 3.92, and 5.35). At the highest aspect ratio, the PANI nanomaterials showed similar results to the bulk PANI materials.

PPY at low concentrations (<9.7 µg/mL) is biocompatible to primary mouse embryonic fibroblasts, the mouse hepatoma cell line, and the human T lymphocyte Jurkat cell line (48), and the cytotoxic effect is dose-dependent [48]. Above 0.291 mg/mL, PPY is significantly toxic for all tested cells. Such information is useful to formulate novel antimicrobial agents for PANI-Zn@CuO and PPY-Zn@CuO pertaining to minimal cytotoxicity, an important topic of future endeavors.

The feasibility of using PANI as an antimicrobial agent has been well reported in the literature. As a cited example, PANI at 0.5% (w/v) or 5 mg/mL is required to suppress the growth of *E. coli* and *S. aureus* [49]. PANI induces the production of hydrogen peroxide, which promotes the formation of hydroxyl radicals, causing biomolecule damage and potentially cell death. Catalase, an enzyme of *E. coli* [50] is capable of scavenging hydrogen peroxide to water and oxygen. Thus, high PANI concentrations are required to ensure the H_2_O_2_ level is over the threshold that can be scavenged, resulting in cellular damage. In the context of cytotoxicity, the PANI amount of 5 mg/mL might cause significant cytotoxicity of fibroblastic cells as discussed earlier. In contrast, the PANI concentration of the PANI-Zn@CuO composite was 0.8 mg/mL, a distinct advantage of its potential use as an antimicrobial agent when the cytotoxicity aspect is taken into consideration. The MIC of the two composites (0.144 mg/mL) was comparable to those of PPY-NT (nanotube)/Ag-NP nanocomposites. In brief, the MIC of PPY-NT (nanotube)/Ag-NP nanocomposites is dependent upon the level of AgNPs. The lowest MIC values are 0.078 and 0.156 mg/mL for the nanocomposites having 15 wt % of Ag-NPs against *E. coli* and *S. aureus*, respectively [51]. Ag-NPs exhibit a spherical shape with an average diameter of 23.12 ± 3.23 nm, and are significantly smaller than the size of the Zn@CuO particles (1–5 µm) in this work.

## 4. Conclusions and Future Possibilities

Two conducting polymer composite materials PANI-Zn@CuO and PPY-Zn@CuO were synthesized using CDs as the only initiator. Zn-doped CuO particles were then decorated on the conducting polymers by simple ultrasonication. The polymer composites exhibited remarkable antimicrobial effects against *E. coli* and *S. aureus*. The mechanism of cell death was mainly attributed to the release of reactive oxygen species (ROS) to impair the bacterial membranes, DNA, and proteins. The adhesion of bacteria to the composites would disrupt the native surface charge of bacterial cells, which induced cell lysis, resulting in eventual cell death. The synthesis of the polymer composites is easy and relatively inexpensive with a broad range of antimicrobial effects against multidrug-resistant bacteria.

The two newly synthesized composites can be extended to other antibiotic-resistant bacteria, depending upon the intended applications and cytotoxicity aspects. Apparently, some potential applications include the fabrication of personal protective equipment (mask, glove, and clothing), bandages, and hygienic products with antimicrobial properties. Other advanced applications encompass the coating of prostheses and medical devices to prevent bacterial infection. In this context, the two polymers must be tested against some priority bacteria and yeast with high antibiotic resistance. Of importance is the incorporation of the two polymer composites in topical cream with antifungal properties against *Candida albicans*, *Candida auris*, and toenail fungus. Candida causes candidiasis in humans, which is acquired in hospitals by patients with weakened immune systems. Toenail fungus, also known as onychomycosis, is a common fungal infection of human toenail by *Trichophyton rubrum* and other fungi. Our polymer composites could be utilized as a “standalone” or an “adjunct” therapy with current antifungal drugs. The two polymer composites are expected to have high efficacy against *T. rubrum* as well as other bacteria and fungi that cause toenail infection. They will also be formulated with some chemicals to soften the infected area and speed up the course of treatment and effectiveness.

Therefore, the cytotoxicity aspect of the polymer composites must also be investigated to assess their safety uses. For external treatment, e.g., topical cream, hand sanitizer, and lacquer, the biocompatibility of the PANI and PPY composites can be conducted using different cell lines, but fibroblasts are the most frequently used line for the determination of cytotoxicity. Cytotoxicity testing is very involved according to the ISO protocol 10,993-5 and several biomarkers are analyzed to decipher the cell death. Of notice is the use of cell-based impedance spectroscopy (ECIS) for the assessment of toxic materials and nanomaterials [52,53]. Cytotoxicity effects on cells are difficult to measure and time-consuming, however, ECIS label-free technology offers quantitative results for cell toxicology and viability in real-time and continuously.

In brief, our paper unravels the synthesis of two new polymer composites, and they are well characterized by different modern equipment. We have also demonstrated a synergic effect by the combination of PPY or PANI with Zn@CuO for inhibiting the growth of *S. aureus* and *E. coli*, two common bacteria, which have been used extensively in the literature. The cytotoxicity aspect of our newly synthesized polymer composites needs to be investigated, depending upon their intended uses.

## Figures and Tables

**Table 1:** The literature on different polymer composites.

Polymer Composite	References
PPY/ZnO/chitosan	[15]
PPY-Ag	[16]
PPY-Ag-NPs	[17]
PPY/dextrin	[18]
PANI/PVA (polyvinyl alcohol)/Ag	[19]
TOCN (TEMPO *-oxidized cellulose nanofibers)/PVA-PPYfilm	[20]
PANI@ZnO	[21]
PANI/Pt-Pd	[22]
PANI/Ag–Pt	[23]
PANI-Ag-Au	[24]

* TEMPO: (2,2,6,6-Tetramethylpiperidin-1-yl) oxyl or (2,2,6,6-tetramethylpiperidin-1-yl) oxidanyl.
